# Ejection fraction, B‐type natriuretic peptide and risk of stroke and acute myocardial infarction among patients with heart failure

**DOI:** 10.1002/clc.23140

**Published:** 2019-01-07

**Authors:** Barry Greenberg, Eric D. Peterson, Jeffrey S. Berger, François Laliberté, Qi Zhao, Guillaume Germain, Dominique Lejeune, Jennifer W. Wu, Patrick Lefebvre, Gregg C. Fonarow

**Affiliations:** ^1^ Division of Cardiovascular Medicine, UC San Diego Health System La Jolla California; ^2^ Division of Cardiology, Duke Clinical Research Institute Durham North Carolina; ^3^ Department of Medicine, New York University School of Medicine New York New York; ^4^ Health Economics & Outcomes Research, Groupe d'analyse Montreal Québec Canada; ^5^ Health Economics and Outcomes Research (HECOR), Cardiovascular, Janssen Scientific Affairs, LLC Trenton New Jersey; ^6^ Department of Medicine, Ronald Reagan‐UCLA Medical Center Los Angeles California

**Keywords:** B‐type natriuretic peptide, ejection fraction, heart failure, myocardial infarction, real‐world, stroke

## Abstract

**Background:**

Real‐world data on the clinical outcomes of heart failure (HF) across the spectrum of ejection fraction (EF) and the prognostic value of B‐type natriuretic peptide (BNP) have not been well examined.

**Hypothesis:**

The real‐world association between the clinical outcomes of HF and EF or BNP levels may differ across different EF or BNP values.

**Methods:**

The Optum Integrated Claims‐Clinical data (07/2009‐09/2016) was used to identify adult patients with ≥1 HF diagnosis during hospitalization or emergency room visit. Three EF cohorts were formed: reduced (rEF; EF < 40%), mid‐range (mrEF; EF 40%‐49%), and preserved EF (pEF; EF ≥ 50%). Stratifications by BNP levels were performed using median BNP as cutoff between high vs low BNP (H‐BNP vs L‐BNP).

**Results:**

In total, 7005 HF patients with EF measurements (2456 patients with both HF and BNP measurements) were identified. rEF patients had higher risk of stroke (hazard ratio [HR] = 1.57, *P* = 0.010) and acute myocardial infarction (AMI) (HR = 2.42, *P* < 0.001) compared to pEF patients. H‐BNP was associated with a significantly higher risk of mortality (*P* < 0.001). rEF patients with H‐BNP had a significantly higher risk of stroke than those with L‐BNP.

**Conclusions:**

Patients with rEF had a significantly higher rate of stroke and AMI vs pEF patients, as did patients with H‐BNP vs L‐BNP. The present study is the first to show the real‐world association of EF and BNP (alone and in combination) with clinical outcomes, further supporting the recommendation to use these markers in clinical practice. These results may help to guide future recommendations and improve the clinical management of HF.

## 1 INTRODUCTION

Heart failure (HF) manifests when the heart's capacity to sustain blood flow is compromised, resulting in shortness of breath, fatigue, and systemic and pulmonary congestion.^1^, ^2^ In 2016, 5.7 million (2.2%) individuals had HF in the United States.^3^, ^4^ The burden of this disease is substantial, and approximately half of patients with HF die from complications ensuing from HF within 5 years following initial diagnosis.^4^ HF patients are also at increased risk of cardiovascular events, including ischemic stroke and acute myocardial infarction (AMI).^3^, ^5^, ^6^ Comorbidities associated with HF, such as diabetes and coronary artery disease (CAD), are risk factors that may be present in a substantial proportion of HF cases.^7^ Current clinical management includes beta‐blockers, angiotensin‐converting enzyme inhibitors, mineralocorticoid receptor antagonists, and/or diuretics to limit fluid accumulation.^1^, ^8^, ^9^

The left‐ventricular ejection fraction (EF) is a measurement of the systolic function of HF patients^10^ that has been shown to predict cardiovascular risks and mortality.^11^ The European Society of Cardiology (ESC) and the American College of Cardiology/American Heart Association Task Force on Clinical Practice Guidelines/Heart Failure Society of America (ACC/AHA/HFSA) recommend using different treatment approaches for HF with reduced (rEF; EF <40%,), mid‐range (mrEF; EF 40%‐49%), and preserved (pEF; EF ≥50%) EF.^1^, ^9^ However, HF diagnosis can be challenging,^12^, ^13^ and the prognostic potential of EF appears to be reduced for values above 40% to 45%,^14^, ^15^ thereby further complicating the diagnosis of HF for patients with mrEF or pEF.^1^, ^12^, ^13^, ^16^ Moreover, the effectiveness of HF‐approved therapies has mainly been demonstrated in rEF patients.^1^ Thus, additional predictors are needed to more accurately stratify HF patients and improve clinical decision making.

B‐type natriuretic peptide (BNP) is a peptide hormone that is now the gold standard diagnostic and prognostic biomarker for HF.^17^ Indeed, a systematic review of 19 studies reported that, for every 100 pg/mL rise in BNP concentration, there is a corresponding 35% increase in the risk of death, and recent updates of the ACC/AHA/HFSA and ESC guidelines recommend using BNP levels in the risk stratification of HF.^1^, ^9^

The use of BNP levels in stratifying HF patients is supported by three prospective studies, which collectively demonstrate that BNP levels correlate with EF,^18^ and that the prognostic value of BNP is equal or even higher than that of EF.^19^, ^20^ However, to the best of our knowledge, the clinical outcomes of HF patients stratified using EF or BNP levels have not been studied in a real‐world setting. Furthermore, there are limited data pertaining to the use of BNP to predict events, such as AMI and ischemic stroke in patients with HF. In order to fill this knowledge gap, this US retrospective claims study was conducted to evaluate the association of cardiovascular events (ie, ischemic stroke, AMI) with EF levels and to assess the prognostic value of BNP in a real‐world setting.

## 2 METHODS

### 2.1 Data source

Data from the Optum's Integrated Claims‐Clinical Database from July 2009 to September 2016 were used. This database includes information on over 10 million US individuals (across 650 hospitals and 6600 clinics) with their adjudicated claims linked to Humedica's electronic medical records. Patient demographics, inpatient and outpatient visits, costs of services, laboratory tests, laboratory results, and mortality data coming from the Social Security Administration Public Death Master File were available. The database was compliant with the Health Insurance Portability and Accountability Act, and, thus, no ethics board review was required.^21^

### 2.2 Study design

A retrospective cohort design was used. Patients with ≥1 HF diagnosis during a hospitalization or emergency room (ER) visit, defined as the index date, were identified from the database.

Included patients were required to have ≥1 primary or secondary HF diagnosis during a hospitalization or ER visit (International Classification of Diseases, Ninth Revision, Clinical Modification [ICD‐9‐CM] codes: 428.xx; ICD‐10‐CM: I50.xx), ≥18 months of continuous enrollment prior to the index date (ie, the baseline period), ≥18 years of age on the index date, and ≥ 1 EF measurement before or after the index date (ie, ±90 days; Appendix S1, Supporting Information). In analyses to evaluate ischemic stroke events, patients with prior stroke or transient ischemic attack (TIA) during the baseline period were excluded. Similarly, patients with a prior AMI during the baseline period were excluded from the analyses evaluating AMI. In stratifications involving BNP levels, HF patients were required to have ≥1 BNP measurement collected during the index hospitalization or ER visit in addition to the EF measurement.

Patients were stratified based on EF, BNP levels, or both (first stratified based on EF, then based on BNP levels). The following EF groups were formed based on EF cutoffs recommended by the ACC/AHA/HFSA and ESC guidelines: rEF (EF <40%), mrEF (EF 40%‐49%), and pEF (EF ≥50%).^1^, ^9^ BNP measurements collected closest to the discharge date were used, and EF measurements collected closest to the index admission date were used to group patients. Stratifications by BNP levels were performed using median BNP levels as a cutoff between low and high BNP (L‐ and H‐BNP, respectively) subgroups (BNP cutoff for: all patients = 411 pg/mL; EF < 40% = 644 pg/mL; EF 40%‐50% = 534 pg/mL; EF ≥ 50% = 321 pg/mL).

### 2.3 Outcome definition

Study outcomes included a primary diagnosis of ischemic stroke or AMI resulting in hospitalization. All‐cause mortality was also evaluated as a secondary outcome to account for the competing risk of death in patients with HF. The observation period was defined as the shortest time frame between the 1‐year period following the index date and the period spanning from the index date up to the earliest date among end of data availability (September 30, 2016), end of insurance coverage, or death. Of note, for the all‐cause mortality analyses, the end of the eligibility period was used as a proxy of the date of death in patients indicated as deceased but without an associated date of death. More specifically, in these patients, the date of death was defined as the last day of the month during which end of eligibility occurred.

### 2.4 Sensitivity analyses

Sensitivity analyses were performed for HF patients diagnosed with (a) CAD (hereinafter referred to as the CAD subgroup), (b) diabetes (hereinafter referred to as the diabetes subgroup) during the baseline period, and (c) patients without any diagnosis of atrial fibrillation (AF) during the baseline period or on the index date.

### 2.5 Statistical analysis

Baseline characteristics were evaluated using means, medians, and standard deviations (SDs) for continuous variables, and using frequencies and percentages for categorical variables. Kaplan‐Meier (KM) rates and log‐rank tests evaluated during the observation period were used to compare the cumulative incidence of study outcomes (ie, stroke and AMI) among the different EF cohorts and BNP subgroups. Adjusted hazard ratios (HRs) and 95% confidence intervals (CIs) were calculated at 12 months using Cox proportional hazards models (ie, time‐to‐event analysis) adjusting for the following covariates evaluated during 18‐month baseline period: age, gender, region, race, insurance type, year of index date, baseline hospitalizations, AF, Quan‐Charlon comorbidity index (Quan‐CCI) score, and CHA_2_DS_2_‐VASc score (Appendx S3).

## 3 RESULTS

### 3.1 Baseline characteristics

A total of 7005 HF patients with an EF measurement were identified, including 1622, 1095, and 4288 patients with rEF, mrEF, and pEF, respectively (Table 1 and Appendix S1). The mean duration of the observation period was 254, 266, and 260 days for HF patients with rEF, mrEF, and pEF, respectively (Table 1).

**Table 1:** Patient baseline characteristics

Characteristics	All HF patients	Ejection fraction < 40%			Ejection fraction 40%‐49%			Ejection fraction ≥ 50%		
	(N = 7005)	All	High BNP^a^	Low BNP^a^	All	High BNP^a^	Low BNP^a^	All	High BNP^a^	Low BNP^a^
		(N = 1622)	(N = 325)	(N = 327)	(N = 1095)	(N = 182)	(N = 183)	(N = 4288)	(N = 719)	(N = 720)
Observation period, days, mean ± SD [median]	259.8 ± 130.8 [366]	254.4 ± 133.2 [366]	234.2 ± 141.2 [300]	256.3 ± 128.9 [366]	265.9 ± 131.0 [366]	241.7 ± 139.8 [344]	272.3 ± 128.1 [366]	260.3 ± 129.8 [366]	241.7 ± 133.5 [294]	264.5 ± 130.1 [366]
Ejection fraction measurement										
Value, %, mean ± SD [median]	50.6 ± 15.1 [55]	28.4 ± 7.6 [30]	27.2 ± 7.8 [28]	28.4 ± 7.3 [30]	43.7 ± 3.0 [44]	43.6 ± 2.9 [44]	44.0 ± 3.1 [45]	60.7 ± 6.8 [60]	60.2 ± 7.0 [60]	61.0 ± 6.6 [60]
Demographics^b^										
Age, years, mean ± SD [median]	73.5 ± 12.2 [76]	71.8 ± 12.8 [74]	74.4 ± 12.1 [78]	68.9 ± 13.7 [71]	72.8 ± 12.2 [75]	75.0 ± 11.8 [79]	71.1 ± 12.7 [74]	74.4 ± 11.9 [77]	77.5 ± 10.4 [81]	72.5 ± 12.4 [75]
Gender, female, n (%)	3361 (48.0%)	590 (36.4%)	134 (41.2%)	115 (35.2%)	413 (37.7%)	88 (48.4%)	67 (36.6%)	2358 (55.0%)	446 (62.0%)	404 (56.1%)
Region^b^, n (%)										
Midwest	3522 (50.3%)	831 (51.2%)	176 (54.2%)	176 (53.8%)	537 (49.0%)	103 (56.6%)	105 (57.4%)	2154 (50.2%)	380 (52.9%)	404 (56.1%)
South	1963 (28.0%)	507 (31.3%)	99 (30.5%)	124 (37.9%)	330 (30.1%)	52 (28.6%)	55 (30.1%)	1126 (26.3%)	200 (27.8%)	224 (31.1%)
Northeast	529 (7.6%)	116 (7.2%)	20 (6.2%)	14 (4.3%)	74 (6.8%)	5 (2.7%)	5 (2.7%)	339 (7.9%)	42 (5.8%)	38 (5.3%)
West	817 (11.7%)	122 (7.5%)	13 (4.0%)	5 (1.5%)	126 (11.5%)	17 (9.3%)	12 (6.6%)	569 (13.3%)	80 (11.1%)	37 (5.1%)
Unknown	174 (2.5%)	46 (2.8%)	17 (5.2%)	8 (2.4%)	28 (2.6%)	5 (2.7%)	6 (3.3%)	100 (2.3%)	17 (2.4%)	17 (2.4%)
Insurance type^b^, n (%)										
Medicare	5130 (73.2%)	1118 (68.9%)	247 (76.0%)	204 (62.4%)	762 (69.6%)	141 (77.5%)	114 (62.3%)	3250 (75.8%)	597 (83.0%)	537 (74.6%)
Commercial insurance	1875 (26.8%)	504 (31.1%)	78 (24.0%)	123 (37.6%)	333 (30.4%)	41 (22.5%)	69 (37.7%)	1038 (24.2%)	122 (17.0%)	183 (25.4%)
Comorbidity index scores^c^										
Quan‐CCI, mean ± SD [median]	2.6 ± 2.3 [2]	2.3 ± 2.3 [2]	2.1 ± 2.1 [2]	2.2 ± 2.2 [2]	2.6 ± 2.4 [2]	2.8 ± 2.7 [2]	2.3 ± 2.1 [2]	2.7 ± 2.4 [2]	3.0 ± 2.4 [3]	2.5 ± 2.3 [2]
CHA_2_DS_2_‐VASc score, mean ± SD [median]	3.9 ± 1.9 [4]	3.4 ± 1.9 [3]	3.6 ± 1.9 [4]	3.2 ± 2.0 [3]	3.8 ± 1.8 [4]	4.1 ± 1.7 [4]	3.6 ± 1.9 [4]	4.1 ± 1.8 [4]	4.5 ± 1.7 [4]	3.9 ± 1.8 [4]
HAS‐BLED score, mean ± SD [median]	2.2 ± 1.1 [2]	1.9 ± 1.1 [2]	2.0 ± 1.0 [2]	1.8 ± 1.1 [2]	2.1 ± 1.0 [2]	2.2 ± 1.0 [2]	1.9 ± 1.1 [2]	2.3 ± 1.0 [2]	2.4 ± 1.0 [2]	2.2 ± 1.0 [2]
Atrial fibrillation^c^, n (%)	1561 (22.3%)	276 (17.0%)	48 (14.8%)	57 (17.4%)	261 (23.8%)	32 (17.6%)	51 (27.9%)	1024 (23.9%)	197 (27.4%)	143 (19.9%)
Prior cerebrovascular accident (stroke or transient ischemic attack), n (%)	708 (10.1%)	146 (9.0%)	33 (10.2%)	24 (7.3%)	124 (11.3%)	22 (12.1%)	22 (12.0%)	565 (13.2%)	97 (13.5%)	86 (11.9%)
Stroke	530 (7.6%)	90 (5.5%)	19 (5.8%)	11 (3.4%)	71 (6.5%)	11 (6.0%)	12 (6.6%)	369 (8.6%)	57 (7.9%)	51 (7.1%)
Transient ischemic attack	319 (4.6%)	48 (3.0%)	4 (1.2%)	12 (3.7%)	43 (3.9%)	5 (2.7%)	6 (3.3%)	228 (5.3%)	43 (6.0%)	33 (4.6%)
Prior acute myocardial infarction^c^^,^ d, n (%)	200 (2.9%)	57 (3.5%)	8 (2.5%)	6 (1.8%)	52 (4.7%)	10 (5.5%)	6 (3.3%)	91 (2.1%)	19 (2.6%)	9 (1.3%)
Prior myocardial infarction, n (%)	390 (5.6%)	106 (6.5%)	14 (4.3%)	13 (4.0%)	97 (8.9%)	16 (8.8%)	13 (7.1%)	187 (4.4%)	32 (4.5%)	17 (2.4%)
Baseline anticoagulants^e^, n (%)										
Any oral anticoagulant	868 (12.4%)	155 (9.6%)	34 (10.5%)	38 (11.6%)	158 (14.4%)	15 (8.2%)	39 (21.3%)	555 (12.9%)	110 (15.3%)	83 (11.5%)
Warfarin	700 (10.0%)	135 (8.3%)	32 (9.8%)	34 (10.4%)	126 (11.5%)	13 (7.1%)	30 (16.4%)	439 (10.2%)	92 (12.8%)	58 (8.1%)
Any NOACs	215 (3.1%)	30 (1.8%)	3 (0.9%)	8 (2.4%)	42 (3.8%)	3 (1.6%)	12 (6.6%)	143 (3.3%)	23 (3.2%)	29 (4.0%)
Antiplatelet	746 (10.6%)	147 (9.1%)	35 (10.8%)	22 (6.7%)	148 (13.5%)	28 (15.4%)	20 (10.9%)	451 (10.5%)	86 (12.0%)	80 (11.1%)

Abbreviations: BNP, B‐type natriuretic peptide; HF, heart failure; NOAC, non‐vitamin K antagonist oral anticoagulant.

a High and low BNP determined using the median as cutoff (EF < 40% = 644 pg/mL; EF 40‐49% = 534 pg/mL; EF ≥ 50% = 321 pg/mL).

b Evaluated at the index date.

c Evaluated during the baseline period.

d Primary diagnosis of myocardial infarction during a hospitalization.

A total of 2456 HF patients with both EF and BNP measurements were identified (ie, the overall population), including 652, 365, and 1439 patients in the rEF, mrEF, and pEF cohorts, respectively (Table 1 and Appendix S1). H‐BNP patients were numerically older than L‐BNP patients across all EF cohorts (eg, mean age [years] for rEF: H‐BNP = 74.4, L‐BNP = 68.9; Table 1). Patients in the pEF cohort included a numerically higher proportion of females (rEF = 36.4%, mrEF = 37.7%, pEF = 55.0%; Table 1). Quan‐CCI appeared similar between H‐ and L‐BNP patients in the rEF cohort (eg, mean Quan‐CCI: H‐BNP = 2.1, L‐BNP = 2.2; Table 1 and Appendix S3).

### 3.2 Outcomes of patients stratified by EF (N = 7005)

**Figure 1:**
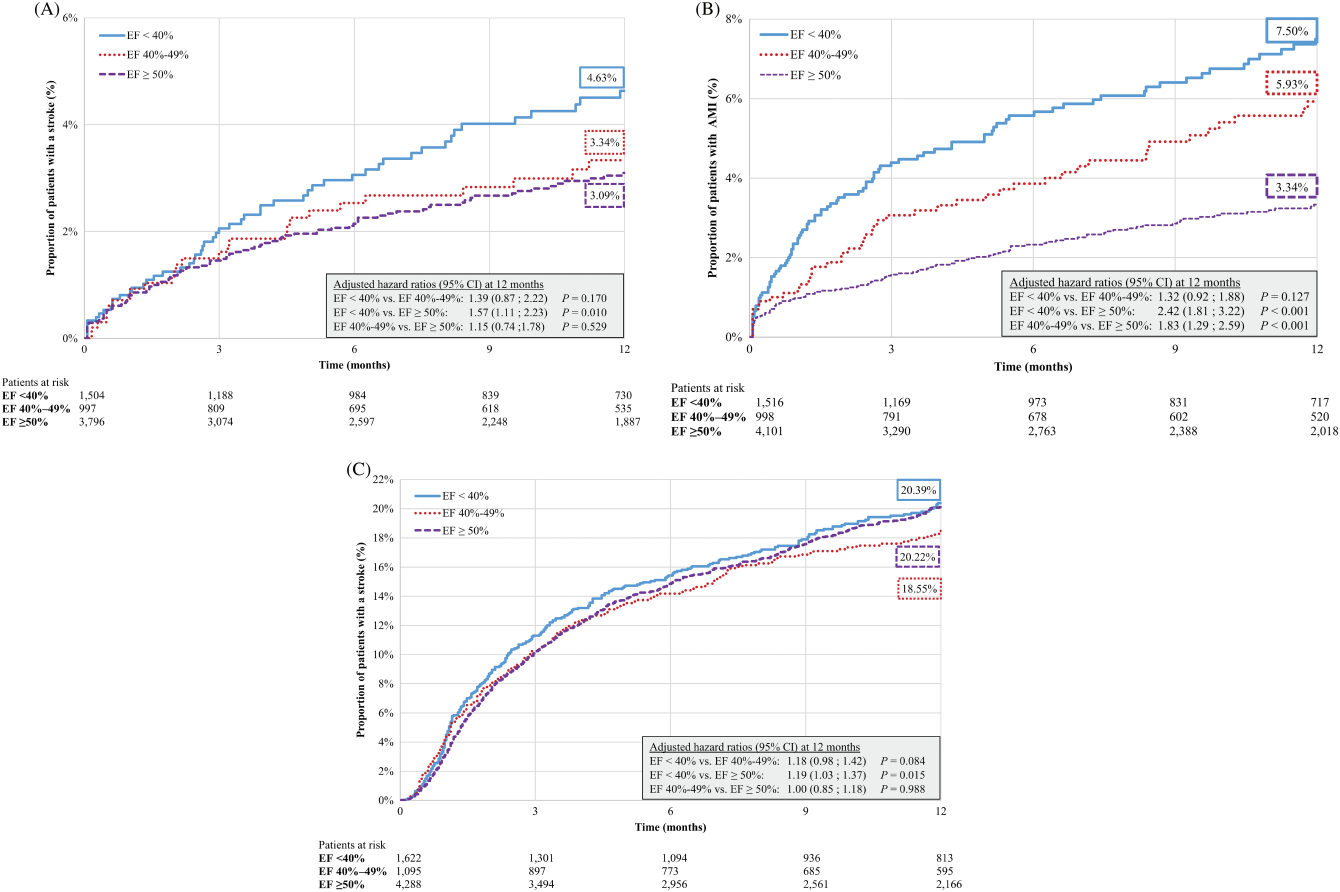
A, Kaplan‐Meier rates of stroke—excluding patients with baseline stroke/trancient ischemic attack (TIA). B, Kaplan‐Meier rates of acute myocardial infarction ‐ excluding patients with baseline AMI, and C, Kaplan‐Meier rates of all‐cause mortality. **The end of the eligibility period was termed the death date for patients indicated deceased without an associated date of death (N = 313)

Patients with rEF had a significant 1.6‐fold higher risk of ischemic stroke compared to patients with pEF during the observation period (ie, HR = 1.57, *P* = 0.010; Figure 1A). The risk of stroke was not significantly different between rEF vs mrEF and between mrEF vs pEF (Figure 1A).

Patients with rEF had a significant 2.4‐fold higher risk of AMI compared to patients with pEF (ie, HR = 2.42, *P* < 0.001; Figure 1B). Similarly, there was not any significant difference in AMI risk between rEF vs mrEF, but there was one between mrEF vs pEF cohorts (ie, HR = 1.83, *P* < 0.001; Figure 1B

Relative to patients with pEF, patients with rEF had a slightly higher risk of all‐cause mortality (ie, HR = 1.19, *P* = 0.015; Figure 1C). Statistical significance was not reached for the rEF vs mrEF or mrEF vs pEF comparisons for this outcome (Figure 1C).

### 3.3 Outcomes of patients stratified by BNP levels (N = 2456)

Independently of EF levels, H‐BNP was not significantly associated with higher risks of ischemic stroke and AMI compared to L‐BNP (Table 2). However, H‐BNP patients had significantly higher risks of all‐cause mortality than L‐BNP patients (ie, HR = 1.40, *P* < 0.001; Table 2).

**Table 2:** Kaplan‐Meier rates and Hazard ratios of cardiovascular events stratified by EF and BNP^a^

Outcomes	Number of patients		Rates (at 1 year) (%)		Log‐rank test *P*‐value	Hazard ratio (95% CI)	*P*‐value
	High BNP	Low BNP	High BNP	Low BNP			
Ischemic stroke							
All EF values	1122	1111	4.09	2.26	0.035	1.63 (0.92‐2.90)	0.095
EF <40%	303	307	7.39	1.25	0.002	5.03 (1.41‐17.92)	0.013
EF 40%‐49%	167	169	4.10	0.61	0.082	6.00 (0.34‐105.10)	0.220
EF ≥50%	640	647	3.35	2.50	0.403	1.32 (0.60‐2.89)	0.492
Acute myocardial infarction							
All EF values	1163	1188	5.72	3.27	0.013	1.56 (0.99‐2.47)	0.056
EF <40%	311	314	6.82	4.92	0.576	1.17 (0.54‐2.52)	0.685
EF 40%‐49%	166	170	6.91	3.64	0.196	1.71 (0.53‐5.56)	0.371
EF ≥50%	687	703	4.22	3.06	0.042	1.27 (0.66‐2.46)	0.475
All‐cause mortality^b^							
All EF values	1228	1228	28.22	18.76	<0.001	1.40 (1.17‐1.67)	<0.001
EF <40%	325	327	27.81	17.90	0.002	1.45 (1.00‐2.10)	0.049
EF 40%‐49%	182	183	25.37	13.65	0.006	1.59 (0.91‐2.77)	0.100
EF ≥50%	719	720	30.79	18.74	<0.001	1.48 (1.17‐1.87)	0.001

BNP, B‐type natriuretic peptide B; EF, ejection fraction.

a High and low BNP determined using the median as cutoff (all = 411 pg/mL; EF < 40% = 644 pg/mL; EF 40%‐50% = 534 pg/mL; EF ≥ 50% = 321 pg/mL).

b The end of the eligibility period was termed the death date if a patient was indicated deceased without an associated date.

### 3.4 Outcomes of patients stratified by EF and BNP levels (N = 2456)

Among rEF patients, the risk of ischemic stroke was significantly higher for H‐BNP patients compared to L‐BNP patients (ie, HR = 5.03, *P* = 0.013; Table 2). Although the risk of ischemic stroke was numerically higher among pEF and mrEF patients with H‐BNP, the differences did not reach statistical significance (*Ps* > 0.05; Table 2). In both the rEF and pEF cohorts, all‐cause mortality was significantly higher for H‐BNP patients compared to L‐BNP patients (ie, pEF mortality: HR = 1.48, *P* = 0.001; Table 2).

### 3.5 CAD and diabetes subgroups

In the CAD subgroup, comparing the rEF to the pEF cohort revealed a significantly higher risk of AMI (ie, HR = 2.21, *P* < 0.001). In sensitivity analyses, all‐cause mortality was also significantly reduced in rEF vs pEF patients (ie, HR = 1.36, *P* < 0.001),. Compared to mrEF, rEF patients had a non‐significantly higher risk for all study outcomes (*Ps* > 0.05; Appendix S2). Although a significantly higher risk of AMI was observed when comparing patients with mrEF and pEF (ie, HR = 1.69, *P* = 0.011; Appendix S2), the same comparison did not reach statistical significance for other outcomes. Similar results were generally observed when comparing the outcomes among the different EF groups of HF patients with diabetes (Appendix S2).

### 3.6 Outcomes of patients without AF

When comparing the cardiovascular outcomes of HF patients without AF, similar conclusions could be drawn. Patients with rEF had a significantly higher risk of stroke (ie, HR [95% CI] = 1.69 [1.10; 2.59], *P* = 0.016), AMI (ie, HR [95% CI] = 2.91 [2.08; 4.06], *P* < 0.001), and mortality (ie, HR [95% CI] = 1.24 [1.03; 1.49], *P* = 0.023) compared to patients with pEF. For patients with either CAD or diabetes, the inclusion or exclusion of AF patients did not have a significant impact on the outcomes stratified by EF levels (data not shown). When stratifying patients either by BNP level alone, or by EF and BNP levels, the magnitudes and directionalities of the differences between patients with low vs high BNP were largely preserved, although statistical significance was lost for some of them (data not shown).

## 4 DISCUSSION

Here, the association between HF clinical outcomes vs EF and BNP levels was investigated, and the prognosis of patients stratified by these variables was evaluated. HF patients with rEF had a significantly higher risk of all evaluated cardiovascular outcomes compared with patients with pEF. Moreover, higher BNP levels were associated with a significantly higher risk of mortality among patients with BNP and EF measurements. Among rEF patients, HF patients with H‐BNP had a significantly greater risk of stroke and all‐cause mortality compared to patients with L‐BNP. Among patients diagnosed with CAD or diabetes, comparing the clinical outcomes across the EF cohorts revealed trends that were largely similar to those observed for the overall population of HF patients, confirming the value of EF in these subpopulations.

Despite EF being widely used in clinical practice for HF risk stratification, conflicting results have been reported about the clinical outcomes of rEF vs pEF patients. In clinical trial settings, the risk of mortality,^14^, ^22^, ^23^ fatal myocardial infarction, and stroke^14^ seem to negatively correlate with EF. Similarly, a meta‐analysis conducted by Somaratne et al suggested that mortality was higher among rEF patients compared with pEF patients.^24^ However, Bhatia et al reported that mortality rates were similar between HF patients with pEF and rEF in a hospital setting.^25^ Results from the present study support the view that rEF is associated with a worse prognosis than pEF since rEF patients were found to have higher risks of all study outcomes compared to pEF patients.

The 2016 ESC guidelines stress the importance of gathering more data about HF patients with mrEF. In fact, virtually all therapies that have been shown to improve outcomes in HF patients are based on trial results in patients with rEF, with little evidence on the effectiveness of these therapies in other populations.^1^ Moreover, EF was reported to have reduced prognostic potential for values above 40% to 45%,^14^ which further complicates clinical decisions for these patients. The results presented in the present study contribute to documenting the prognosis of mrEF patients. Indeed, mrEF patients were found to have a higher risk of AMI compared to patients with pEF, but no such difference was observed when comparing the risk of ischemic stroke between these two cohorts, which might be attributed to the limited predictive power of EF for these EF values.^14^, ^15^

The ESC guidelines also highlight challenges inherent to the diagnosis and treatment of HF patients with pEF.^1^ It has been reported that the prevalence of HF with pEF is substantial (~50%) and seemingly increases over time.^26^ Thus, predictors other than EF may be instrumental to help physicians make informed clinical decisions for this population as well. In line with this concept, BNP levels were found to be significant predictors of mortality in the present study. However, neither the risk of stroke nor that of AMI were significantly different when comparing H‐BNP and L‐BNP patients. This might be in part attributable to the smaller sample sizes for analyses performed with a BNP‐based stratification compared to those performed with an EF‐based stratification, although other factors may contribute. Interestingly, comparing stroke and AMI risk across all EF cohorts among patients also diagnosed with diabetes or CAD revealed trends that were very similar to those observed in the overall HF population, suggesting that conclusions on the prognostic value of EF hold true for these patients. In addition, as might be expected, the rates of AMI appeared higher in patients with CAD compared with the overall population, although no statistical test was performed. This might indicate that patients with CAD and HF (HF/CAD) have unmet needs that are not addressed by the current medications.

The most recent guidelines do not endorse anticoagulation treatment in HF^1^, ^9^ largely because of disappointing results from clinical trials that assessed the efficacy and safety profile of warfarin vs aspirin,^27^, ^28^, ^29^, ^30^ although these data remain controversial.^31^ To date, the use of non‐vitamin K oral antagonist (NOACs) is only approved for HF patients also diagnosed with AF, but the impact of NOACs on HF patients who also suffer from other cardiovascular conditions, such as CAD, remains unknown. The data from the present study suggest that HF patients without AF also have unmet needs, which may help in guiding the design of future studies and trials.

### 4.1 Limitations

The present study is subject to a number of limitations. First, due to the inherent nature of insurance claims databases, coding inaccuracies or omissions in procedures and diagnoses could have occurred. Second, despite adjusting for many baseline covariates, the impact of unmeasured confounders cannot be ruled out. Third, this analysis does not differentiate between the etiologies and heterogeneity of HF, which could play a role in the clinical outcomes and patient care. Fourth, although the closest value to the index date was used to define EF, this measure was allowed to be recorded up to 90 days before or after the index date, a time frame during which EF may vary. Similarly, the most recent BNP value prior to discharge date of the index hospitalization or ER was used, however, BNP measures could also vary throughout the same hospitalization or ER visit. Finally, the requirement for diagnostic tests like EF and/or BNP may lead to a selection bias for patients with characteristics that may be different from the overall population of patients with HF.

## 5 CONCLUSION

The prognostic value of EF and BNP levels among HF patients remains understudied in the real world. In this retrospective cohort study that used data from a large US insurance claims database, HF patients with rEF were found to have significantly worse clinical outcomes compared to patients with pEF, thereby confirming the reliability of EF in this subpopulation. BNP levels were also a reliable predictor of mortality. Moreover, the prognosis of rEF patients was significantly worse among those who had high BNP levels with respect to stroke risk, and mortality. The present work also contributes to documenting the prognosis of mrEF patients, who were found to have a higher risk of AMI vs pEF patients. This study is the first to show the real‐world association of EF and BNP with clinical outcomes, further supporting the recommendation to use these markers in clinical practice. These results may help guide future recommendations and improve the clinical management of HF.

## CONFLICTS OF INTEREST

One of the authors (Qi Zhao) is an employee of Janssen Scientific Affairs. Five of the authors (François Laliberté, Guillaume Germain, Dominique Lejeune, Jennifer W. Wu, and Patrick Lefebvre) are employees of Groupe d'analyze, Ltée, a consulting company that has received research grants from Janssen Scientific Affairs. Barry Greenberg, Eric D. Peterson, Jeffrey S. Berger, and Gregg Fonarow have received research grants from Janssen Scientific Affairs. Jeffrey S. Berger has received research funding from AstraZeneca.

## Supporting information


**APPENDIX S1** Patient selection flowchart

**APPENDIX S2** KM rates and hazard ratios of stroke, acute mi, all‐cause mortality, and composite outcomes by EF Levels at 12 months—HF patients with CAD or Diabetes

**APPENDIX S3** Description of the calculation of the Quan‐CCI, CHA_2_DS_2_‐VASc, and HAS‐BLED scores
