# Supplementary material for: Ejection fraction, B‐type natriuretic peptide and risk of stroke and acute myocardial infarction among patients with heart failure
Source: Clin Cardiol. 2019 Jan 7;42(2):277–84. doi: 10.1002/clc.23140 (PMC6712323; doi:10.1002/clc.23140)
Supplement: Supplementary file 1 — APPENDIX S1 Patient selection flowchart [file CLC-42-277-s002.docx]

**Appendix A: Patient selection flowchart**

|  | **Optum Integrated from July 2009** | | | |
| --- | --- | --- | --- | --- |
|  | Patients with ≥1 HF^1^ diagnosis | | | |
|  | **N = 120,036** | | | |
|  |  | | | |
|  | ≥18 months of continuous eligibility prior to index date **(newly diagnosed HF patients)** | | | |
|  | **N = 69,333 (57.8%)** | | | |
|  |  | | | |
|  | At least 18 years of age as of the index date | | | |
|  | **N = 69,104 (99.7%)** | | | |
|  |  | | | |
|  | HF documented during a hospitalization or an emergency room visit | | | |
|  | **N = 36,681 (53.1%)** | | | |
|  |  | | | |
|  | With EMR activity within a window of ±90 days around the index date | | | |
|  | **N = 25,254 (68.8%)** | | | |
|  |  | | | |
| **EF-based stratification** | With EF value^2^: |  |  | **N = 7,005 (27.7%)** |
|  | EF <40%: |  |  | **N = 1,622** |
|  | EF 40%-49%: |  |  | **N = 1,095** |
|  | EF ≥50%: |  |  | **N = 4,288** |
|  |  |  |  |  |
| **BNP-based stratification** | With BNP value^3^: |  |  | **N = 2,456 (35.1%)** |
|  | EF <40%: |  |  | **N = 652** |
|  | EF 40%-49%: |  |  | **N = 365** |
|  | EF ≥50%: |  |  | **N = 1,439** |

**Abbreviations:** BNP = B-type natriuretic peptide; EF=ejection fraction; EMR=electronic medical record; HF=heart failure

**Notes:**

1. Primary or secondary diagnosis with ICD-9-CM codes: 428.x, and ICD-10-CM code: I50.x.
2. Based on the closest EF value to the index date within a window of ±90 days around the index date.

Based on the BNP value collected during the index hospitalization or emergency room visit
