# Supplementary material for: Ejection fraction, B‐type natriuretic peptide and risk of stroke and acute myocardial infarction among patients with heart failure
Source: Clin Cardiol. 2019 Jan 7;42(2):277–84. doi: 10.1002/clc.23140 (PMC6712323; doi:10.1002/clc.23140)
Supplement: Supplementary file 2 — APPENDIX S2 KM rates and hazard ratios of stroke, acute mi, all‐cause mortality, and composite outcomes by EF Levels at 12 months—HF patients with CAD or Diabetes [file CLC-42-277-s001.docx]

**Appendix B -** KM Rates and Hazard Ratios of Stroke, Acute MI, All-Cause Mortality, and Composite Outcomes by EF Levels at 12 Months - HF Patients with CAD or Diabetes

|  |  |  | **Survival function (Kaplan-Meier estimates)** | | |  | **HR (95% CI)^1^** | | | | | | | |
| --- | --- | --- | --- | --- | --- | --- | --- | --- | --- | --- | --- | --- | --- | --- |
| **EF cohorts comparisons** | |  | **<40%**  **(rEF)** | **40%-49%**  **(bEF)** | **≥50%**  **(pEF)** |  | **<40% vs. 40%-49%**  **(rEF vs. bEF)** | **P-value** |  | **<40% vs. ≥50%**  **(rEF vs. pEF)** | **P-value** |  | **40%-49%**  **vs. ≥50%**  **(bEF vs. pEF)** | **P-value^1^** |
|  |  |  |  |  |  |  |  |  |  |  |  |  |  |  |
|  |  |  |  |  |  |  |  |  |  |  |  |  |  |  |
| **CAD** | **Ischemic stroke^2^** | **N** | **960** | **652** | **1,826** |  | 1.09 (0.59–1.99) | 0.784 |  | 1.27 (0.79–2.05) | 0.323 |  | 1.06 (0.60–1.87) | 0.840 |
|  |  | **%** | 4.25% | 3.28% | 3.02% |  |  |  |  |  |  |  |  |  |
|  | **AMI^3^** | **N** | **940** | **628** | **1,906** |  | 1.39 (0.93–2.06) | 0.106 |  | 2.21 (1.59–3.08) | < 0.001 |  | 1.69 (1.13–2.53) | 0.011 |
|  |  | **%** | 10.44% | 7.50% | 4.80% |  |  |  |  |  |  |  |  |  |
|  | **All-cause mortality^4^** | **N** | **1,046** | **725** | **2,093** |  | 1.21 (0.96–1.52) | 0.101 |  | 1.36 (1.14–1.63) | < 0.001 |  | 1.09 (0.89–1.35) | 0.406 |
|  |  | **%** | 22.12% | 19.49% | 19.36% |  |  |  |  |  |  |  |  |  |
| **Diabetes** | **Ischemic stroke^2^** | **N** | **480** | **359** | **1,349** |  | 0.86 (0.45–1.64) | 0.647 |  | 1.95 (1.10–3.44) | 0.022 |  | 2.14 (1.20–3.83) | 0.010 |
|  |  | **%** | 5.61% | 5.97% | 3.20% |  |  |  |  |  |  |  |  |  |
|  | **AMI^3^** | **N** | **490** | **353** | **1,469** |  | 0.91 (0.54–1.53) | 0.720 |  | 2.09 (1.34–3.26) | 0.001 |  | 2.09 (1.29–3.38) | 0.003 |
|  |  | **%** | 8.06% | 9.05% | 4.66% |  |  |  |  |  |  |  |  |  |
|  | **All-cause mortality^4^** | **N** | **534** | **402** | **1,552** |  | 1.14 (0.84–1.55) | 0.386 |  | 1.22 (0.97–1.54) | 0.088 |  | 1.08 (0.83–1.40) | 0.583 |
|  |  | **%** | 22.06% | 20.23% | 21.09% |  |  |  |  |  |  |  |  |  |

**Abbreviations:** AMI=acute myocardial infarction; ; bEF=borderline ejection fraction; CAD=coronary artery disease; EF=ejection fraction; HR=hazard ratio; KM=Kaplan-Meier; pEF=preserved ejection fraction; rEF=reduced ejection fraction

**Notes:**

1. Calculated using a Cox proportional hazard model adjusting for age, gender, region, race, insurance type, year of index date, baseline hospitalizations, atrial fibrillation, CCI, CHA_2_DS_2_-VASc.
2. Excluding patients with a ischemic stroke/TIA during the baseline period.
3. Excluding patients with a myocardial infarction during the baseline period.
4. The end of the eligibility period was termed as the death date if a patient was indicated deceased without an associated date.
