# Supplementary material for: Ejection fraction, B‐type natriuretic peptide and risk of stroke and acute myocardial infarction among patients with heart failure
Source: Clin Cardiol. 2019 Jan 7;42(2):277–84. doi: 10.1002/clc.23140 (PMC6712323; doi:10.1002/clc.23140)
Supplement: Supplementary file 3 — APPENDIX S3 Description of the calculation of the Quan‐CCI, CHA2DS2‐VASc, and HAS‐BLED scores [file CLC-42-277-s003.docx]

**Appendix C:** Description of the calculation of the Quan-CCI, CHA_2_DS_2_-VASc, and HAS-BLED scores

1. The Quan-Charlson comorbidity index (Quan-CCI) is a score that predicts in-hospital mortality over a one year period based on the presence of Charlson comorbidities (see list below). It can be calculated by summing up the weights assigned to each Charlson comorbidity present in a given patient.

| **Charlson comorbidities** | **Weight^1^** |
| --- | --- |
| Myocardial infarction | 0 |
| Congestive heart failure | 2 |
| Peripheral vascular disease | 0 |
| Cerebrovascular disease | 0 |
| Dementia | 2 |
| Chronic pulmonary disease | 1 |
| Rheumatologic disease | 1 |
| Peptic ulcer disease | 0 |
| Hemiplegia or paraplegia | 2 |
| Renal disease | 1 |
| AIDS/HIV | 4 |
| Liver disease |  |
| Mild^2^ | 2 |
| Moderate or severe^2^ | 4 |
| Diabetes |  |
| Without chronic complications^3^ | 0 |
| With chronic complications^3^ | 1 |
| Malignancy |  |
| Any, including leukemia and lymphoma^4^ | 2 |
| Metastatic solid tumor^4^ | 6 |
| **Maximum score** | 24 |

**Abbreviations:** AIDS = acquired immune deficiency syndrome; HIV = human immunodeficiency virus

**Notes:**

1. Quan et al. 2011, Am J Epidemiol 173(6): 676-82
2. Mild and Moderate or severe liver disease are mutually exclusive Charlson comorbidities
3. Diabetes with and without chronic complications are mutually exclusive Charlson comorbidities
4. Any malignancy and metastatic solid tumors are mutually exclusive Charlson comorbidities
5. CHA_2_DS_2_-VASc is a score that predicts the risk of stroke and thromboembolism within a one year period based on the risk factors listed below. It can be calculated by summing up the weights assigned to each risk factor present in a given patient.

| **Accronym** | **Risk Factor** | **Weight^1^** |
| --- | --- | --- |
| C | Congestive heart failure/LV dysfunction | 1 |
| H | Hypertension | 1 |
| A | Age ≥75 years^2^ | 2 |
| D | Diabetes mellitus | 1 |
| S | Stroke/TIA/TE | 2 |
| V | Vascular disease^3^ | 1 |
| A | Age 65-74 years^2^ | 1 |
| S | Sex category (i.e., female gender) | 1 |
|  | **Maximum score** | 9 |

**Abbreviations:** LV = left ventricular; TE = thromboembolism; TIA = transient ischemic attack

**Notes:**

1. Lip et al. 2010. Chest. 137 (2): 263-72
2. Age ≥75 and age 65-74 are mutually exclusive risk factors
3. Includes prior myocardial infarction, peripheral artery disease, or aortic plaque
4. HAS-BLED is a score that predicts the risk of major bleeding within a one year period based on the risk factors listed in the table below. It can be calculated by summing up the weights assigned to each risk factor present in a given patient.

| **Accronym** | **Risk Factor** | **Weight^1^** |
| --- | --- | --- |
| H | Hypertension | 1 |
| A | Abnormal renal or liver function (1 point each) | 1 or 2 |
| S | Stroke | 1 |
| B | Bleeding | 1 |
| L | Labile INRs | 1 |
| E | Elderly (i.e., >65 years) | 1 |
| D | Drug or alcohol (1 point each) | 1 or 2 |
|  | **Maximum score** | 9 |

**Abbreviations**: INR = international normalized ratio

**Notes:**

1. Pisters et al. 2010. Chest. 138 (5): 1093-100
